# Innate Immune Responses of Vaccinees Determine Early Neutralizing Antibody Production After ChAdOx1nCoV-19 Vaccination

**DOI:** 10.3389/fimmu.2022.807454

**Published:** 2022-01-25

**Authors:** Ching-Fen Shen, Chia-Liang Yen, Yi-Chen Fu, Chao-Min Cheng, Tzu-Chi Shen, Pei-De Chang, Kuang-Hsiung Cheng, Ching-Chuan Liu, Yu-Tzu Chang, Po-Lin Chen, Wen-Chien Ko, Chi-Chang Shieh

**Affiliations:** ^1^ Institute of Clinical Medicine, College of Medicine, National Cheng Kung University, Tainan City, Taiwan; ^2^ Department of Pediatrics, National Cheng Kung University Hospital, College of Medicine, National Cheng Kung University, Tainan City, Taiwan; ^3^ Institute of Biomedical Engineering, National Tsing Hua University, Hsinchu City, Taiwan; ^4^ Department of Pathology, National Cheng Kung University Hospital, College of Medicine, National Cheng Kung University, Tainan City, Taiwan; ^5^ Department of Internal Medicine, National Cheng Kung University Hospital, College of Medicine, National Cheng Kung University, Tainan City, Taiwan

**Keywords:** innate immune, neutralizing antibodies, SARS-CoV-2 vaccines, adenoviral vector vaccine, immunosenescence

## Abstract

**Background:**

Innate immunity, armed with pattern recognition receptors including Toll-like receptors (TLR), is critical for immune cell activation and the connection to anti-microbial adaptive immunity. However, information regarding the impact of age on the innate immunity in response to SARS-CoV2 adenovirus vector vaccines and its association with specific immune responses remains scarce.

**Methods:**

Fifteen subjects between 25-35 years (the young group) and five subjects between 60-70 years (the older adult group) were enrolled before ChAdOx1 nCoV-19 (AZD1222) vaccination. We determined activation markers and cytokine production of monocyte, natural killer (NK) cells and B cells *ex vivo* stimulated with TLR agonist (poly (I:C) for TLR3; LPS for TLR4; imiquimod for TLR7; CpG for TLR9) before vaccination and 3-5 days after each jab with flow cytometry. Anti-SARS-CoV2 neutralization antibody titers (surrogate virus neutralization tests, sVNTs) were measured using serum collected 2 months after the first jab and one month after full vaccination.

**Results:**

The older adult vaccinees had weaker vaccine-induced sVNTs than young vaccinees after 1^st^ jab (47.2±19.3% vs. 21.2±22.2%, *p* value<0.05), but this difference became insignificant after the 2^nd^ jab. Imiquimod, LPS and CpG strongly induced CD86 expression in IgD^+^CD27^-^ naïve and IgD^-^CD27^+^ memory B cells in the young group. In contrast, only the IgD^+^ CD27^-^ naïve B cells responded to these TLR agonists in the older adult group. Imiquimode strongly induced the CD86 expression in CD14^+^ monocytes in the young group but not in the older adult group. After vaccination, the young group had significantly higher IFN-γ expression in CD3^-^ CD56^dim^ NK cells after the 1^st^ jab, whilst the older adult group had significantly higher IFN-γ and granzyme B expression in CD56^bright^ NK cells after the 2^nd^ jab (all *p* value <0.05). The IFN-γ expression in CD56^dim^ and CD56^bright^ NK cells after the first vaccination and CD86 expression in CD14^+^ monocyte and IgD^-^CD27^-^double-negative B cells after LPS and imiquimod stimulation correlated with vaccine-induced antibody responses.

**Conclusions:**

The innate immune responses after the first vaccination correlated with the neutralizing antibody production. Older people may have defective innate immune responses by TLR stimulation and weak or delayed innate immune activation profile after vaccination compared with young people.

## Background

With global spreading of SARS-CoV-2, COVID-19 pandemic has caused tremendous impact on the entire world with fast-transmitted illness and huge loss of lives. Rapid surge of severe COVID-19 infections not only threatened infected people, but also overloaded medical resources and led to collapse of healthcare system in many countries. The destructive impact of this pandemic on economic, sociological, and psychological aspect is overwhelming. Vaccines for the prevention of SARS-CoV-2 infection promise to be the fastest and most effective way to control this pandemic. Up to now, there are several COVID-19 vaccines available for clinical use and more are under developments. Among them, the chimpanzee non-replicating adenovirus vector vaccine, ChAdOx1 nCoV-19 (AZD1222) developed at Oxford University and produced by AstraZeneca is one of the most widespread used vaccines around the world (1). This AstraZeneca (AZ) vaccine was reported to have an overall 70.4% vaccine efficacy against symptomatic disease after two does and 100% of vaccine efficacy against severe COVID-19 infection and hospitalization in an early clinical study (2). Even with this high overall protective effect, the protection efficiency in different individuals may vary significantly. Importantly, the efficacy of COVID-19 vaccine in people of different age groups is not well studied, even though older adults is the most important risk factor for developing severe COVID-19 disease (3, 4).

To generate adequate immunity after vaccination, early innate immune responses are crucial for subsequent signaling for T cell activation and adaptive immune development. Innate immunity is triggered *via* different pathways in the different formulations of novel vaccines to induce immunity against SARS-CoV-2 infection. For mRNA vaccine, the endosomal Toll-like receptor (TLR3 and TLR7) bind to single-strand RNA (ssRNA) in the endosome, while component in the inflammasome including MDA5, RIG-1, NOD2 and PKR binds to ssRNA and double-stranded RNA (dsRNA) in the cytosol, all together leading to cellar activation and production of inflammatory mediators (5). For the adenovirus vector vaccine (AdV), it contains self-adjuvanticity properties because the vector’s hexon protein itself is an intrinsic adjuvant to stimulate innate immune responses (6). Following injection, the innate immune recognition by AdV particles involved multiple pattern-recognition receptors, such as Toll-like receptor 3 (TLR3), TLR7/8, and in particular TLR9 to recognize dsDNA, ssRNA and ssDNA of the viral vector. In antigen presenting cells including dendritic cells (DCs) and macrophages, these innate immune stimulations subsequently trigger the production of type I interferon (IFN), multiple proinflammatory cytokine and chemokines. These stimulated immune cells may express high levels of co-stimulatory molecules to stimulate T cells in draining lymph nodes where further activation of adaptive immune cells including B cells occurs (7).

Aging is often associated with important immunological alterations including changes in number of innate and adaptive immune cells and different responses to immune stimulations, leading to different of immune functions, termed immunosenescence (8, 9). Changes in innate immunity with aging includes reduced chemotaxis, aberrant cytokine production, and weakened TLR signaling (10). This impairment in innate immunity then affects the capacity to process and present antigen to T cells and activate B cells, hence weakens adaptive immunity. Immunosenescence has been increasingly considered a major drawback for vaccine-induced immune response. The age-associated decrease in TLR function in human DCs has been linked with poor antibody response to influenza immunization, showing the importance of innate immune system in vaccine response and the influence of aging (11). It’s very likely that the immunosenescence of the older adults will lead to no response or sub-optimal response to vaccination, a potential risk for breakthrough infection when encountering the defense against new SARS-CoV2 variants. Recently, the emergence of two major variants of concern (VOCs), the Delta (B.1.617.2) and Omicron (B.1.1.529) variants have raised the concern that antibody generated by two doses of COVID-19 vaccines are insufficient for protection against infection. The neutralization activity after two doses of COVID-19 vaccine were found to be lower against these two VOCs in comparison with previous strains. In the real world, the vaccine effectiveness also decreased greatly. The effectiveness of AZ vaccine decreased from 74.5% for Alpha variant to 67.0% for Delta variant while the effectiveness of BNT162b2 vaccine lowered from 93.7% for Alpha variant to 88.0% for Delta variant (12). For Omicron variants, the decrease of protection was even more obvious (13).

Several approaches had been adapted to boost immune response in the elderly, including adding more potent adjuvants, changing the route of administration, increasing the dose of immunogen, or changing the vaccine composition with more immunogenic target (14). High-dose inactivated influenza vaccine which contains four-times hemagglutinin antigen than the standard influenza vaccine is available for people older than 65 years old (15). In addition, vaccine adjuvants, including TLR agonists or oil-in-water emulsion (MF59 and AS03 in influenza vaccine, and AS02 in recombinant herpes zoster vaccine), which evoke stronger antigen presenting cells activation and proinflammatory cytokines production, have been used in vaccines for older people (16, 17). Recently, scientists had proposed a new concept of system vaccinology incorporating the concepts of immunobiography integrated with clinical, immunological and “omics” data to identify biomarkers to guide the precise development of vaccines for different population groups (18). Therefore, there is an urgent need to elucidate the innate immune response among vaccinees of different age populations and the relationship between innate immune responses and the protection effect after vaccination. In the current study, we investigated peripheral blood immune cell activation and cytokine secretion induced by TLR stimulation or AZ vaccine in older and young age groups and measured the anti-SARS-CoV-2 spike protein RBD antibody and neutralization antibody after vaccination to identify the role of innate immunity in vaccine-induced protection.

## Methods

### Volunteer Vaccination Study Design

Healthy adults within two age groups (25-35 years and 60-70 years) without any contra-indications for vaccine and pre-existing immunocompromised conditions were eligible for this study. Participants provided written informed consent upon recruitment. The protocol of this study was reviewed and approved by the Institutional Review Board (IRB) of National Cheng Kung University Hospital (NCKU) (IRB no. A-BR-110-051). Participants with preceding immunocompromised status, receiving cytotoxic treatment, or immunosuppressants were excluded. All participants received two doses of AstraZeneca (AZ) SARS-CoV-2 vaccines with 8 to 12 weeks apart. Blood samples were taken at 4 time points (before vaccination, 3-5 days after both jabs and 1 month after full vaccination). Participants’ demographic data, clinical response after vaccination were recorded for further analysis.

### Innate Immune Cell Activation

#### Sample Processing

Peripheral blood mononuclear cells (PBMCs) were isolated with Ficoll-Paque from heparinized whole blood samples collected from young and older adult subjects before and 3 days after vaccination. Isolated PBMCs were suspended with RPMI1640+10% FBS. Cells isolated from subjects before first vaccination were stimulated with TLR agonists including poly(I:C) (20 μg/ml, for TLR3), lipopolysaccharides (LPS, 10 μg/ml, for TLR4), imiquimod (10 μg/ml, for TLR7) and CpG (20 ng/ml, for TLR9). All the TLR agonists were from InvivoGen (San Diego, CA, USA, product information in **Supplementary Table 1**). Stimulated cells were cultured at 37°C for 3 days. On day 2, stimulated cells were treated with protein transport inhibitor brefeldin A (BFA). Cells isolated from vaccinated subjects were treated with brefeldin A (BFA) immediately. Cells isolated from vaccinated subjects after vaccination were treated with brefeldin A (BFA) immediately without TLR-agonist stimulations then harvested one day after.

#### Surface Markers and Intracellular Cytokines Analysis With Flow Cytometry

BFA-treated PBMCs were washed twice with iced cold phosphate-buffered saline (PBS). Cells were incubated with 10% of fetal bovine serum (FBS) for 30 minutes. After blocking with 10% FBS, antibodies against surface markers of NK, B cells, and monocytes were used. For detecting NK cells, anti-CD3 APC, anti-CD56 FITC, and anti- CD16 APC/Cy7 were used. CD56^dim^ NK is CD3^-^, CD16^+^ and CD56^low^ and CD56^bright^ NK is CD3^-^, CD16^-^ and CD56^high^ in lymphocyte region. For detecting B cells, anti-CD19 FITC, anti-IgD PE/Cy7, and anti-CD27 APC/Cy7 were used. Naïve B cell is CD19^+^, IgD^+^ and CD27^-^, double negative B cell is CD19^+^, IgD^-^ and CD27^-^, Unswitched memory cell is CD19^+^, IgD^+^ and CD27^+^, switched memory B cell is CD19^+^, IgD^-^ and CD27^+^. For detecting monocytes, anti-CD14 APC/Cy7, anti-CD68 PE/Cy7, anti-CD86 BB700 and anti-CD204 BV421 were used. All the staining monoclonal antibody were from BD Biosciences (San Jose, CA, USA, product information in **Supplementary Table 1**). After incubating with antibodies on ice for 30 minutes, cells were washed twice with staining buffer containing 2% FBS in PBS. Cells were fixed with 4% paraformaldehyde for 15 minutes and washed twice with iced cold PBS. Cell permeabilization were performed with a BD permeabilization kit. In brief, fixed cells were incubated with 1x BD Perm/Wash solution on ice for 15 minutes. After being washed with Perm/Wash solution, the cells were incubated with antibodies against intracellular proteins including anti-IFN-γ PE/Cy7, anti-IFN-α PE, anti-IL-10 APC anti-IL-6 PE, and anti-granzyme B BB700 on ice for 30 minutes. After wash twice with 1x BD Perm/Wash solution, stained cells were analyzed with FACS Canto II flow cytometry. Data was analyzed with FlowJo™ v10 software (TreeStar).

### SARS-CoV-2 Antibody Assay

#### Surrogate SARS-CoV-2 Neutralization Test (sVNT)

The cPass SARS-CoV-2 Neutralization Antibody Detection Kit (GenScript, Piscataway, NJ) was performed according to the manufacturer’s instructions (19). Briefly, patient sera were mixed with sample dilution buffer (1:10) and horseradish peroxidase conjugated recombinant SARS-CoV-2 receptor binding domain (HRP-RBD) fragments. The pre-incubation step allows for the binding of circulating neutralizing to the HRP-RBD. After 30 minutes at 37°C, the mixture will be added to a capture plate which had been precoated with the ACE2 protein. Any unbound HRP-RBD or HRP-RBD bound to non-neutralization antibodies was bound to the plate while the circulating neutralization antibody HRP-RBD complexes remained in the supernatant and was removed during the wash step. After washing, tetramethyl benzidine substrate solution was added followed by the Stop Solution which quenched the reaction, turning the well color yellow. The plates were immediately read at 450 nm on a microtiter plate reader.

Signal inhibition was calculated as follows:

Percent Signal Inhibition=(1-average optical density value of a sample/average optic density value of negative control)*100%

The test results were interpreted as positive when the percent signal inhibition was ≥30%, which is the cut-off for signal inhibition claimed by the manufacturer.

#### Anti-SARS-CoV-2 Total Antibody (Roche Elecsys)

Roche Elecsys anti-SARS-CoV-2 S (Roche Diagnostics; hereafter called Roche S), an automated electrochemiluminescence immunoassay for the quantitative determination of pan-immunoglobulin to RBD of the spike protein of SARS-CoV-2 was used. The assay was performed on Roche cobas e601 system (Roche Diagnostics) as described before and used plasma or serum from vaccinated volunteers for measurement (20).

### Statistical Analysis

Descriptive analyses of numerical variables were presented as mean and standardized deviation (Mean ± SD), and categorical variables were presented as frequency and percentage. Means of continuous variables were assessed with the Student’s t test, Wilcoxon signed-rank and 1-way ANOVA. A *P*-value of< 0.05 was considered statistical significance. All statistical analyses were performed using Prism software (GraphPad Software).

## Results

### Older Adult Vaccinees Had Distinct Immune Cell Composition Proportions and Weakened First-Dose Antibody Response

**Figure 1:**
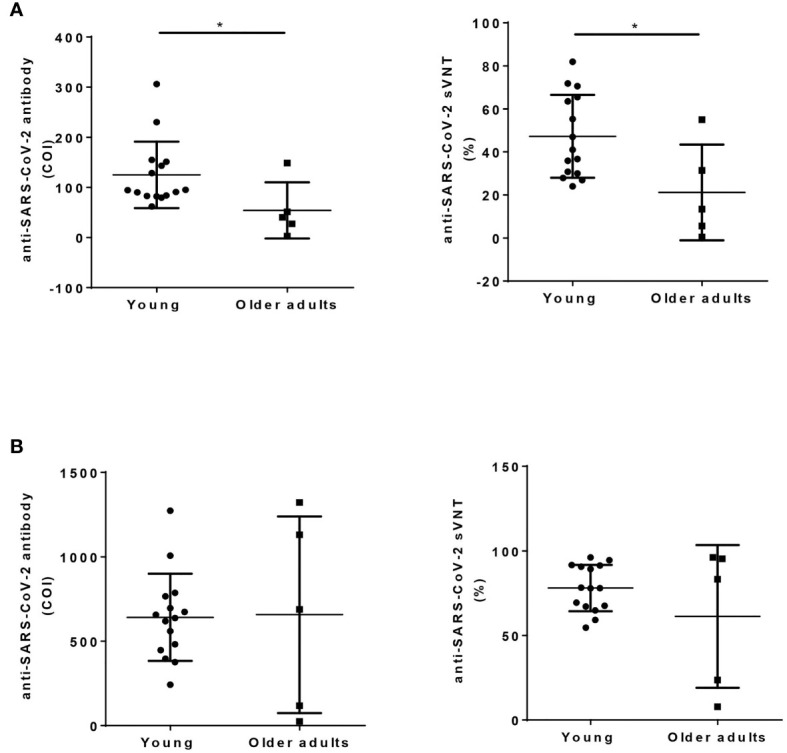
Humoral response after ChAdOx1 nCoV-19 vaccination in young and older adult groups. Anti-SARS-CoV-2 total antibody (left panel, Roche Elecsys), and sVNT (right panel, cPass) were measured using serum collected 2 months after the first dose of vaccination **(A)**, and 1 month after the 2^nd^ dose of vaccination **(B)** in young and older adult groups. Statistical significance was determined using t-test between 2 groups. (*p value < 0.05).

A total of 15 young and 5 older adult people were recruited into this study with mean ages of 29.9 and 66.4 years, respectively. Before vaccination, these two groups did not differ in baseline hemogragm, including total white blood cells, platelet counts, or hemoglobin level (**Table 1**). However, the older adult vaccinees had less neutrophils, higher lymphocytes, higher CD56^+^ NK cell, and CD19^+^ B cells compared to young vaccines (**Table 1**, all *p* value < 0.05). The older adult vaccinees had weaker 1^st^ dose antibody response than young vaccinees (sVNT: 21.2 ± 22.2% vs. 47.2 ± 19.3%, *p* value <0.05). However, both groups demonstrated good booster response after the 2^nd^ jab and had no statistically different antibody levels even though some vaccinees in the older adult group still had low antibody levels (**Figure 1**).

**Table 1:** Demographic information, baseline hemogram, immune cells distribution and antibody response post vaccination of the vaccinees by age group.

Group	Young vaccinees (n=15)	Older adult vaccinees (n=5)	*p* value
Age	29.9 ± 7.3	66.4 ± 1.9	**<0.001**
Male/Female ratio	1.5	4	0.786
Baseline hemogram			
WBC (x k/cmm)	6720 ± 2144	5540 ± 720	0.29
Hb (g/dl)	13.9 ± 0.9	14.1 ± 0.9	.66
Platelet (x k/cmm)	239.3 ± 71.9	225.4 ± 55.4	.74
Neutrophil (%)	63.9 ± 5.7	50.6 ± 5.5	**< 0.01**
Lymphocyte (%)	26.02 ± 4.4	37.5 ± 5.3	**< 0.01**
NK cells/lymphocytes (%)	12.1 ± 5.8	22.1 ± 8.1	**0.0073**
Monocyte (%)	7.2 ± 1.3	8.0 ± 1.5	.37
Post-vaccination antibody			
2 months after 1^nd^ dose			
Anti-SARS-CoV-2 Ab (Roche, cutoff index, COI)	125.0 ± 66.3	54.1 ± 55.9	**0.046**
Anti-SARS-CoV-2 sVNT (%)	47.2 ± 19.3	21.2 ± 22.2	**0.02**
1 month after 2^nd^ dose			
Anti-SARS-CoV-2 Ab (Roche, cutoff index, COI)	641.2 ± 258.3	657.0 ± 582.6	0.956
Anti-SARS-CoV2 sVNT (%)	78.0 ± 13.7	61.3 ± 42.2	0.181

Data are shown as mean ± standard deviation.

Numbers in bold indicates statistical significance (p value < 0.05).

### Aberrant TLR-Induced Monocyte and B Cell Activations in PBMCs of Older Adult Subjects

**Figure 2:**
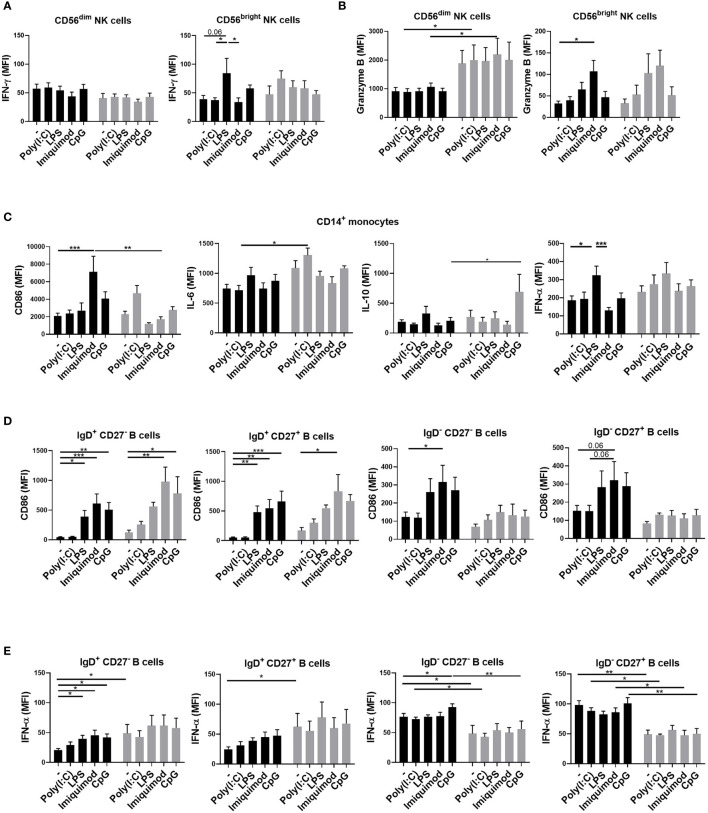
Aberrant TLR-induced monocyte and B cell activation in the older adult subjects. PBMCs were isolated from young (black bars) and older adult subjects (gray bars) before vaccination. TLR-induced IFN-γ **(A)** and granzyme B **(B)** expression in CD3^-^CD56^dim^ or CD3^-^CD56^bright^NK cells; CD86, IL-6, IL-10, and IFN-α in monocytes **(C)**; CD86 **(D)** and IFN-α **(E)** in B cells of the young and older adult vaccinees were detected after 3 days of stimulation. Statistical significance was determined using ANOVA with Tukey’s multiple-comparisons testing between all groups. (Black: young vaccinees; Grey: older adult vaccinees) (*p value < 0.05; **p value <0.01; ***p value < 0.001).

We collected blood samples before and 3 days after the first and second ChAdOx1 nCoV-19 vaccination. Before the vaccination, their PBMCs were first stimulated with TLR agonists and analyzed with flow cytometry. There were no significant differences in the percentages of cytokine-expressing cells after TLR-agonists stimulation in young and older adult groups (data not shown). Among the parameters we examined, no significant differences in the induced expression of IFN-γ and granzyme B levels in NK cells between young and older adult subjects were found (**Figures 2A, B**). However, we found that imiquimod-induced CD86 expression on monocytes was higher in young subjects when compared with that in older adult subjects. (**Figure 2C**). Poly(I:C)-induced IL-6 and CpG-induced IL-10 expressions in monocytes, however, were stronger in the older adult group. (**Figure 2C**). Moreover, all the TLR agonists except poly (I:C) induced significantly higher IFN-α production and CD86 cell-surface expression in IgD^+^CD27^-^ naïve B cells in young and older adult vaccinees but there were no significant differences in TLR-induced CD86 expression in IgD^+^ CD27^-^ naïve B cells and IgD^+^CD27^+^ unswitched memory B cells between young and older adult subjects (**Figure 2D**). TLR-induced CD86 and IFN-α expression levels from IgD^-^CD27^-^ double-negative (DN) B cells and IgD^-^CD27^+^ switched memory B cells were significantly lower in older adult subjects when compared with young subjects (**Figure 2E**). These findings indicated that there are aberrant TLR-induced monocyte and B cell activation in the older adult subjects.

### Delayed NK and Monocyte Activation in the Older Adult Subjects After ChAdOx1 nCoV-19 Vaccination

**Figure 3:**
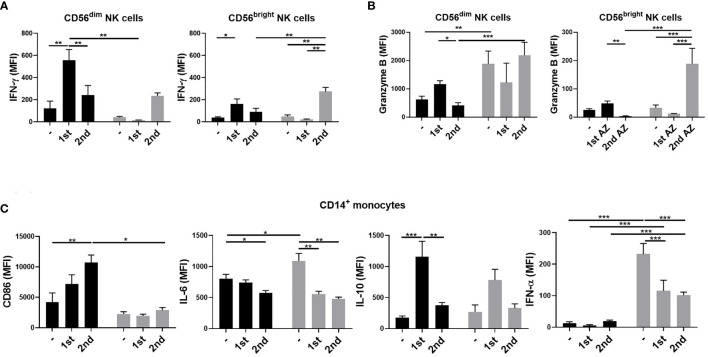
NK cell and monocyte activation 3 days after ChAdOx1 nCoV-19 vaccination. PBMCs were isolated from young (black bars) and older adult vaccinees (gray bars) 3 days after the first and second vaccinations. IFN-γ **(A)** and granzyme B **(B)** expression in CD3^-^CD56^dim^ or CD3^-^CD56^bright^NK cells; CD86, IL-6, IL-10, and IFN-α in monocytes **(C)** of the young and older adult vaccinees were analyzed by flow cytometry. Statistical significance was determined using ANOVA with Tukey’s multiple-comparisons testing between all groups. (Black: young vaccinees; Grey: older adult vaccinees). (*p value < 0.05; **p value <0.01; ***p value < 0.001).

We then investigated the phenotypes of NK and monocyte activation 3 days after vaccinations in the young and older adult groups. We found that the expressions of IFN-γ in CD56^dim^ and CD56^bright^ NK cells were enhanced after the first vaccination in the young group but not in the older adult group (**Figures 3A, B**). However, the expressions of IFN-γ in CD56^dim^ and CD56^bright^ NK cells and granzyme B expression in CD56^bright^ NK cells were enhanced after the second vaccination in the older adult group. We also found that in the older adult group, the percentages of IFN-γ- and granzyme B-producing NKs were reduced after the first vaccination and the percentages restored after the second vaccination (**Supplementary Figures 1A, B**). In monocytes, we found that the cells isolated from the older adult subjects produced significantly lower levels of CD86 when compared with young subjects, who had higher and increasing CD86 expression levels after the 1^st^ and the 2^nd^ vaccinations. (**Figure 3C**). Moreover, the IL-6 and IFN-α levels in monocytes and the percentage of IL-6-producing monocytes were significantly higher in the older adult group before vaccination but decreased after the 1^st^ and 2^nd^ vaccination. However, there were no significant changes in the percentages of IFN-α-expressing monocytes between young and older adult groups after vaccinations (**Supplementary Figure 1C**). We also found that the IL-10 levels in monocytes were enhanced after the first vaccination in both young and older adult groups. The percentages of IL-10-expressing monocytes in young subjects were enhanced after the first and second vaccination. However, the percentage of IL-10-expressing monocytes was lowered in the older adults after the second vaccination. There were no significant differences between young and older adult groups regarding the expression of CD86 and IFN-α 3 days after the 1^st^ and 2^nd^ jabs on B cells. (data not shown).

### Stronger NK and Monocyte Activation After the First Vaccination and TLR-Induced DN B Cell Activation in Subjects With Higher Early Antibody Levels

**Figure 4:**
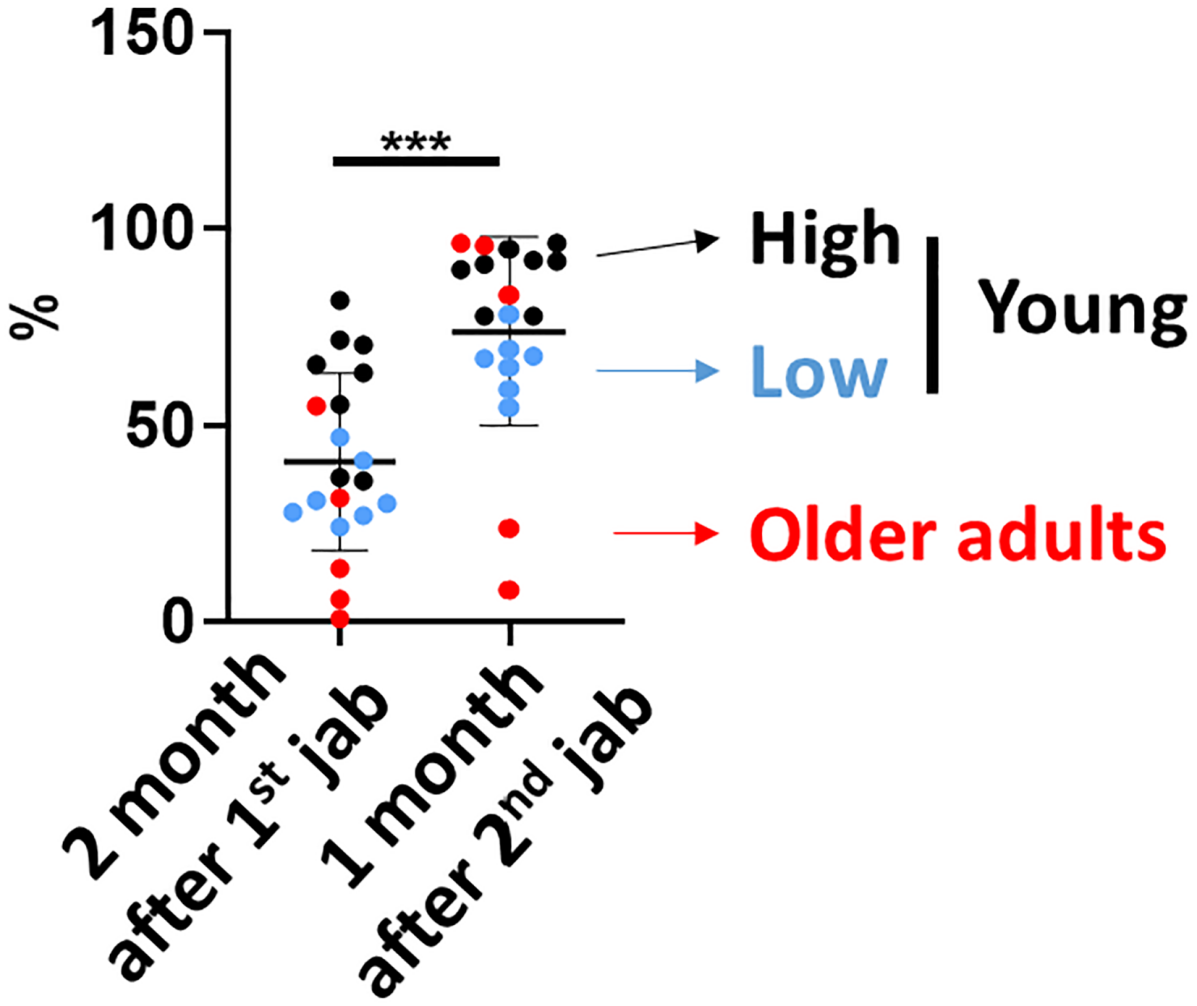
Neutralization antibody titers 2 months after the first vaccination correlated with neutralization antibody titers 1 month after the second vaccination. The SARS-CoV-2 surrogate virus neutralization antibody titers (sVNT) were measured 2 months after the first vaccination and 1 month after the second vaccination. The high sVNT, low sVNT and older adult group all demonstrated higher sVNT 1 month after the second vaccination than 2 months after first vaccination. Statistical significance was determined using Wilcoxon signed rank test between 2 groups. (***p value < 0.001).

**Figure 5:**
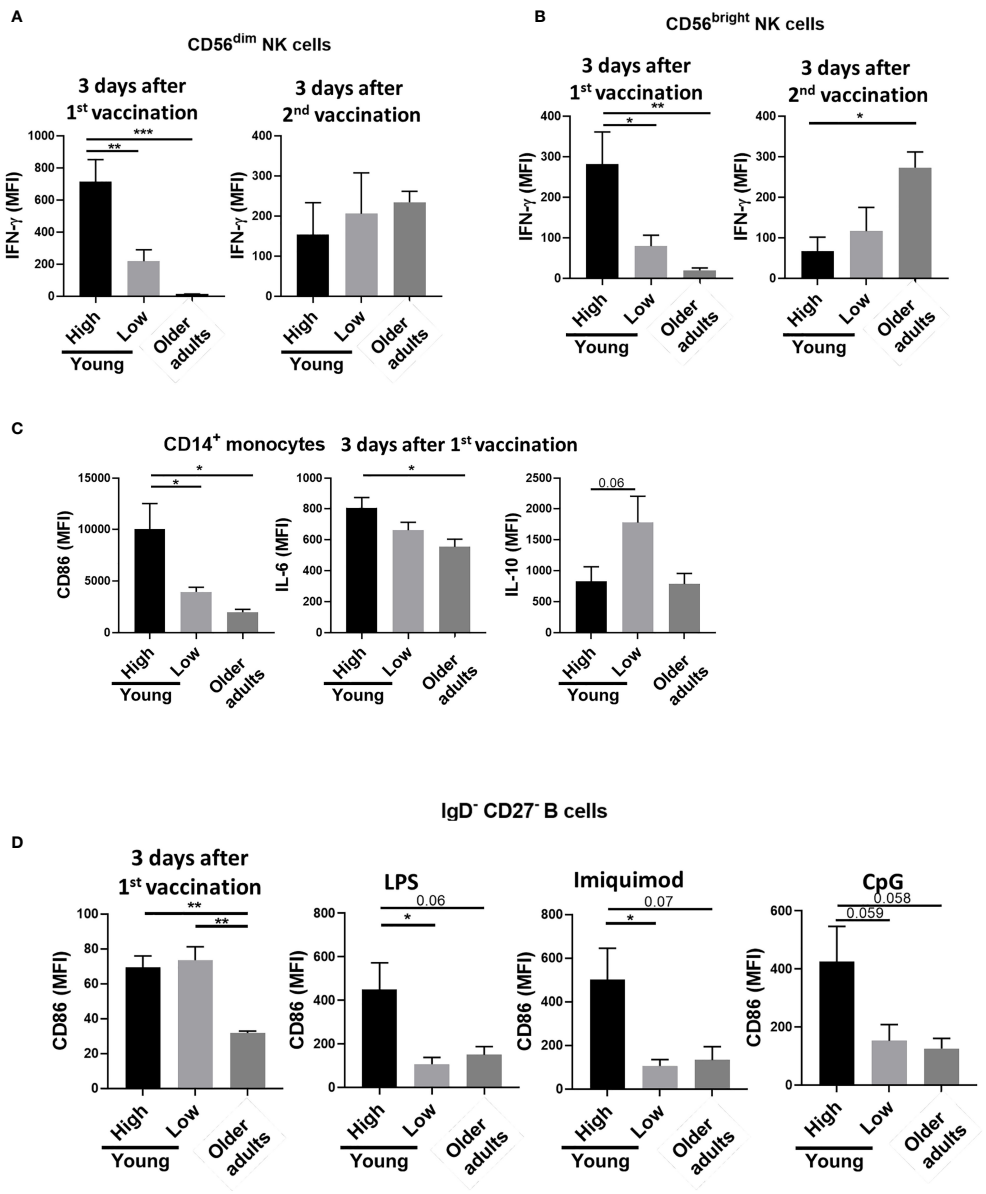
Stronger NK and monocyte activation after the first vaccination and TLR-induced IgD-CD27-double-negative (DN) B cell activation in subjects with higher early antibody levels IFN-γ expressions in CD3^-^CD56^dim^ **(A)** or CD3^-^CD56^bright^ **(B)** NK cells; CD86, IL-6, and IL-10 expression in monocytes **(C)** 3 days after the first vaccination were compared between high and low sVNT groups of the young vaccinees and the older adult vaccinees. **(D)** CD86 expression 3 days after the first vaccination and CD86 levels after LPS, imiquimod, and CpG stimulation were compared between high and low sVNT groups. Statistical significance was determined using ANOVA with Tukey’s multiple-comparisons testing between all groups. (*p value < 0.05; ** *p* value < 0.01; ***p value < 0.001).

We next investigated whether the innate immune activation correlated with neutralization antibody titers induced by ChAdOx1 nCoV-19 vaccination. We compared the sVNT 2 months after the 1^st^ vaccination and 1 month after the second vaccination from the whole cohort simultaneously. We found that subjects whose early levels of sVNT were higher tended to have higher sVNT 1 month after the second vaccination (**Figure 4**). Therefore, we divided the young subjects into high (black) and low groups (blue) according to the sVNT. The high sVNT, low sVNT and older adult group all demonstrated significant booster effect and had higher sVNT one month after full vaccination than two months after 1^st^ jab (all *p* value < 0.05). Next, we compared the innate immune activation between high and low sVNT groups and the older adult subjects. We found that IFN-γ productions in CD56^dim^ and CD56^bright^ NK cells were higher in the high sVNT group after the first vaccination. The IFN-γ productions in CD56^dim^ and CD56^bright^ were lower in both older adult subjects and young subjects with lower sVNT. However, there were no differences in IFN-γ productions in CD56^dim^ NK cells after the second vaccination and IFN-γ productions in CD56^bright^ NK cells were higher when compared with young subjects with higher sVNT (**Figures 5A, B**). We also found that monocyte CD86 expressions were higher in the high sVNT group after the first vaccination. Meanwhile, anti-inflammatory cytokine IL-10 expressions from young monocytes of the lower sVNT group were higher (**Figure 5C**). Moreover, we found that DN CD86 expression was significantly lower in older adult subjects 3 days after 2^nd^ vaccination (**Figure 5D**). Moreover, LPS- and imiquimod-induced DN B cells had higher CD86 expressions in the high sVNT group when compared with subjects with lower sVNT and older adult subjects.

## Discussion

In the present study, we showed that young and older vaccinees had different baseline immune cell distribution and activities. Older adult subjects had diminished TLR-induced monocyte and B cell activation compared to young subjects. The immune cells activation and intracellular cytokine expression following vaccination are different in older and young vaccinees. Importantly, we found that TLR-induced B cell activation and first-dose vaccination-induced and NK and monocyte activation correlate with the vaccine-induced neutralization antibody levels.

TLRs had been proved to be pivotal immune activators which bridges the innate and adaptive immunity. SARS-CoV-2 was known to trigger the innate immune system through TLRs 3, 7, and 8 during the early infection. The dysfunction of TLRs has been reported to be associated with severe COVID cases (21). Aging process comes with several immunological changes described as immunosenscence and inflammaging as two significant aspects of immune dysfunction in older adult people (9). Some immune functions weaken with aging (immunosenescence) while other inflammatory activity may become stronger when people become old (inflammaging). In our experiments, innate immune cells of young vaccinees responded more strongly and more efficiently to TLR agonist stimulation, especially more B cell activation either in CD86 expression or IFN-α production, and more monocyte with CD86 expression when compared to older vaccinees, reflecting the immunosenscence in older adult people. The older adult group had notably higher IFN-γ and granzyme B expression in CD56^bright^ NK cells after the 2^nd^ jab (**Figures 3A, B**), representing the inflammaging which may be too late to induce early protective antibody production.

NK cells are innate lymphoid cells that respond rapidly during primary infection and have adaptive characteristics enabling them to integrate innate and acquired immune responses. They are recently recognized as a key regulator of vaccine-elicited T and B cell responses and memory cells that contribute to pathogen control. This critical role of NK cell activation in vaccine-induced immunity is demonstrated in vaccination against pathogens including influenza, yellow fever, and tuberculosis (22). A previous study examining the draining lymph nodes after influenza vaccination showed that NK cells are recruited regional lymph nodes and activated by type I IFNs produced by LN macrophages. The activated NK cells subsequently produced IFN-γ, which in turn regulates the recruitment of IL-6^+^ CD11b^+^ dendritic cells (23). Actually, NK cells make both early and sustained IFN-γ responses after vaccination and represent over 70% of all IFN-γ-secreting cells (24). The activation of NK cells is critical of IL-2-secreting effector memory T cells and overall vaccine-induced response. Therefore, researchers proposed using the assays NK cell IFN-γ production, and NK cytotoxicity as the tool for evaluating correlates of vaccine-induced immunity (25). Our study demonstrated that IFN-γ expressions in NK cells after 1^st^ jab correlated with SARS-CoV-2 vaccine-induced neutralizing antibody, which echoes previous findings. Since NK cells may play essential roles in developing efficacious vaccine-induced protection, there are discussions about NK cell-mediated modulation of the immune response and its implication on immunization strategies and the development of next-generation vaccines (26, 27).

Naïve B cells differentiate to antibody-producing plasma cells after vaccine stimulation to produce protective antibodies. The DN B cells, previously characterized as unconventional memory B cells with negative expression of both CD27 and surface IgD, were detected in healthy individuals at low levels within peripheral blood and tonsils but are expanded in peripheral blood of older adult patients with systemic lupus erythematosus (28, 29). The role of DN B cells in the humoral immune response remains unclear, but recent research has demonstrated their proinflammatory ability in autoimmune disease and protective ability following vaccination. In the present study, we demonstrated that the degree of CD86 expression of DN B cells after LPS or imiquimod stimulation positively correlated with vaccine induced neutralization antibody. This suggests that DN B cells might constitute a significant transient population during the B cell maturation process, and its activation by innate stimulation may set the stage for subsequent vaccine-induced antibody production.

## Conclusion

Although the changes in the immune system in the older adults have been studied in recent years, the effects of aging on the innate immune activation and consequently unresponsiveness to novel COVID-19 vaccination are still unknown (9). We first found that TLR-induced monocyte and B cell activation was dampened in the older adult subjects. Different activation and intracellular protein expression profiles induced by different TLR agonists may provide further information about the critical point, which may lead to abnormal vaccine responses in older adults. We also found that TLR-induced B cell activation and the first vaccination-induced NK and monocyte activations were related to the neutralization antibody elicited by the ChAdOx1 nCoV-19 vaccination. Therefore, innate immune activation is crucial for the successful activation of protective humoral immunity during vaccination, especially with the wildly adopted viral vector vaccines (30).

## Data Availability Statement

The raw data supporting the conclusions of this article will be made available by the authors, without undue reservation.

## Ethics Statement

The studies involving human participants were reviewed and approved by Institutional Review Board (IRB) of National Cheng Kung University Hospital (NCKU). The patients/participants provided their written informed consent to participate in this study.

## Author Contributions

C-FS and C-CS are the guarantor of the content of this manuscript, had full access to all of the data in the study and take responsibility for the integrity of the data. C-FS, C-LY, and C-CS contributed to the study conception and design, collection of data, data analysis and interpretation, and critical review of the manuscript. C-CL, P-LC and C-MC contributed to the data analysis, and critical review of the manuscript. W-CK contributed to interpretation, critical review of the manuscript. All authors contributed to the article and approved the submitted version.

## Funding

This work was supported by the Clinical Medical Research Center, National Cheng Kung University Hospital, Taiwan (NCKUH-11002017, NCKUH-11009002, NCKUH-11102024) and Ministry of Science and Technology, Taiwan (110-2923-B-006-001-MY4).

## Conflict of Interest

The authors declare that the research was conducted in the absence of any commercial or financial relationships that could be construed as a potential conflict of interest.

## Publisher’s Note

All claims expressed in this article are solely those of the authors and do not necessarily represent those of their affiliated organizations, or those of the publisher, the editors and the reviewers. Any product that may be evaluated in this article, or claim that may be made by its manufacturer, is not guaranteed or endorsed by the publisher.

## Supplementary Material

Supplementary Figure 1 Cytokine-producing NK and monocyte 3 days after ChAdOx1 nCoV-19 vaccination. PBMCs were isolated from young (black bars) and older adult vaccinees (gray bars) 3 days after the first and second vaccinations. The percentage of IFN-γ- **(A)** and granzyme B- **(B)** expressing cells in CD3-CD56dim or CD3-CD56brightNK cells; IL-6-, IL-10-, and IFN-α-expressing monocytes **(C)** of the young and older adult vaccinees were analyzed by flow cytometry. Statistical significance was determined using ANOVA with Tukey’s multiple-comparisons testing between all groups. (Black: young vaccinees; Grey: older adult vaccinees). (*p value < 0.05; **p value < 0.01; ***p value < 0.001).

## References

[1] Our World in Data. Coronavirus Pandemic (COVID-19) (2020). Available at: https://ourworldindata.org/coronavirus (Accessed October 13, 2021).

[2] Voysey M Clemens SAC Madhi SA Weckx LY Folegatti PM Aley PK. Safety and Efficacy of the ChAdOx1 Ncov-19 Vaccine (AZD1222) Against SARS-CoV-2: An Interim Analysis of Four Randomised Controlled Trials in Brazil, South Africa, and the UK. Lancet (2021) 397(10269):99–111. doi: 10.1016/S0140-6736(20)32661-1 33306989 PMC7723445

[3] Soiza RL Scicluna C Thomson EC. Efficacy and Safety of COVID-19 Vaccines in Older People. Age Ageing (2021) 50(2):279–83. doi: 10.1093/ageing/afaa274 PMC7799251 33320183

[4] Ramasamy MN Minassian AM Ewer KJ Flaxman AL Folegatti PM Owens DR. Safety and Immunogenicity of ChAdOx1 Ncov-19 Vaccine Administered in a Prime-Boost Regimen in Young and Old Adults (COV002): A Single-Blind, Randomised, Controlled, Phase 2/3 Trial. Lancet (2021) 396(10267):1979–93. doi: 10.1016/S0140-6736(20)32466-1 PMC7674972 33220855

[5] Pardi N Hogan MJ Porter FW Weissman D. mRNA Vaccines—A New Era in Vaccinology. Nat Rev Drug Discov (2018) 17(4):261–79. doi: 10.1038/nrd.2017.243 PMC5906799 29326426

[6] Hartman ZC Appledorn DM Amalfitano A. Adenovirus Vector Induced Innate Immune Responses: Impact Upon Efficacy and Toxicity in Gene Therapy and Vaccine Applications. Virus Res (2008) 132(1-2):1–14. doi: 10.1016/j.virusres.2007.10.005 18036698 PMC4039020

[7] Teijaro JR Farber DL. COVID-19 Vaccines: Modes of Immune Activation and Future Challenges. Nat Rev Immunol (2021) 21(4):195–7. doi: 10.1038/s41577-021-00526-x PMC7934118 33674759

[8] Connors J Bell MR Marcy J Kutzler M Haddad EK. The Impact of Immuno-Aging on SARS-CoV-2 Vaccine Development. Geroscience (2021) 43(1):31–51. doi: 10.1007/s11357-021-00323-3 33569701 PMC7875765

[9] Pietrobon AJ Teixeira FME Sato MN. I Mmunosenescence and Inflammaging: Risk Factors of Severe COVID-19 in Older People. Front Immunol (2020) 11:579220. doi: 10.3389/fimmu.2020.579220 33193377 PMC7656138

[10] Pereira B Xu XN Akbar AN. Targeting Inflammation and Immunosenescence to Improve Vaccine Responses in the Elderly. Front Immunol (2020) 11:583019. doi: 10.3389/fimmu.2020.583019 33178213 PMC7592394

[11] Panda A Qian F Mohanty S van Duin D Newman FK Zhang L. Age-Associated Decrease in TLR Function in Primary Human Dendritic Cells Predicts Influenza Vaccine Response. J Immunol (2010) 184(5):2518–27. doi: 10.4049/jimmunol.0901022 PMC3867271 20100933

[12] Lopez Bernal J Andrews N Gower C Gallagher E Simmons R Thelwall S. Effectiveness of Covid-19 Vaccines Against the B. 1.617. 2 (Delta) Variant. N Engl J Med (2021) 38(7):585–94. doi: 10.1056/NEJMoa2108891 PMC8314739 34289274

[13] Andrews N Stowe J Kirsebom F Toffa S Rickeard T Gallagher E. Effectiveness of COVID-19 Vaccines Against the Omicron (B. 1.1. 529) Variant of Concern. MedRxiv (2021) 2021.12.14.21267615. doi: 10.1101/2021.12.14.21267615

[14] Lefebvre JS Haynes L. Vaccine Strategies to Enhance Immune Responses in the Aged. Curr Opin Immunol (2013) 25(4):523–8. doi: 10.1016/j.coi.2013.05.014 PMC3775954 23764092

[15] DiazGranados CA Dunning AJ Kimmel M Kirby D Treanor J Collins A. Efficacy of High-Dose Versus Standard-Dose Influenza Vaccine in Older Adults. N Engl J Med (2014) 371(7):635–45. doi: 10.1056/NEJMoa1315727 25119609

[16] O’Hagan DT Ott GS Nest GV Rappuoli R Giudice GD. The History of MF59^®^ Adjuvant: A Phoenix That Arose From the Ashes. Expert Rev Vaccines (2013) 12(1):13–30. doi: 10.1586/erv.12.140 23256736

[17] Cunningham AL Garçon N Leo O Friedland LR Strugnell R Laupèze B. Vaccine Development: From Concept to Early Clinical Testing. Vaccine (2016) 34(52):6655–64. doi: 10.1016/j.vaccine.2016.10.016 27769596

[18] Ciabattini A Nardini C Santoro F Garagnani P Franceschi C Medaglini D. Vaccination in the Elderly: The Challenge of Immune Changes With Aging. Semin Immunol (2018) 40:83–94. doi: 10.1016/j.smim.2018.10.010 30501873

[19] Tan CW Chia WN Qin X Liu P Chen MI Tiu C. A SARS-CoV-2 Surrogate Virus Neutralization Test Based on Antibody-Mediated Blockage of ACE2-Spike Protein-Protein Interaction. Nat Biotechnol (2020) 38(9):1073–8. doi: 10.1038/s41587-020-0631-z 32704169

[20] Jung K Shin S Nam M Hong YJ Roh EY Park KU. Performance Evaluation of Three Automated Quantitative Immunoassays and Their Correlation With a Surrogate Virus Neutralization Test in Coronavirus Disease 19 Patients and Pre-Pandemic Controls. J Clin Lab Anal (2021) 35(9):e23921. doi: 10.1002/jcla.23921 34369009 PMC8418513

[21] Menezes MCS Veiga ADM Martins de Lima T Kunimi Kubo Ariga S Vieira Barbeiro H de Lucena Moreira C. Lower Peripheral Blood Toll-Like Receptor 3 Expression Is Associated With an Unfavorable Outcome in Severe COVID-19 Patients. Sci Rep (2021) 11(1):15223. doi: 10.1038/s41598-021-94624-4 34315957 PMC8316546

[22] Wagstaffe HR Mooney JP Riley EM Goodier MR. Vaccinating for Natural Killer Cell Effector Functions. Clin Transl Immunol (2018) 7(1):e1010. doi: 10.1002/cti2.1010 PMC5822400 29484187

[23] Farsakoglu Y Palomino-Segura M Latino I Zanaga S Chatziandreou N Pizzagalli DU. Influenza Vaccination Induces NK-Cell-Mediated Type-II IFN Response That Regulates Humoral Immunity in an IL-6-Dependent Manner. Cell Rep (2019) 26(9):2307–15.e5. doi: 10.1016/j.celrep.2019.01.104 30811982

[24] Long BR Michaelsson J Loo CP Ballan WM Vu BA Hecht FM. Elevated Frequency of Gamma Interferon-Producing NK Cells in Healthy Adults Vaccinated Against Influenza Virus. Clin Vaccine Immunol (2008) 15(1):120–30. doi: 10.1128/CVI.00357-07 PMC2223854 18003818

[25] Horowitz A Behrens RH Okell L Fooks AR Riley EM. NK Cells as Effectors of Acquired Immune Responses: Effector CD4+ T Cell-Dependent Activation of NK Cells Following Vaccination. J Immunol (2010) 185(5):2808–18. doi: 10.4049/jimmunol.1000844 20679529

[26] Rydyznski CE Waggoner SN. Boosting Vaccine Efficacy the Natural (Killer) Way. Trends Immunol (2015) 36(9):536–46. doi: 10.1016/j.it.2015.07.004 PMC4567442 26272882

[27] Cox A Cevik H Feldman HA Canaday LM Lakes N Waggoner SN. Targeting Natural Killer Cells to Enhance Vaccine Responses. Trends Pharmacol Sci (2021) 42(9):789–801. doi: 10.1016/j.tips.2021.06.004 34311992 PMC8364504

[28] Wei C Anolik J Cappione A Zheng B Pugh-Bernard A Brooks J. A New Population of Cells Lacking Expression of CD27 Represents a Notable Component of the B Cell Memory Compartment in Systemic Lupus Erythematosus. J Immunol (2007) 178(10):6624–33. doi: 10.4049/jimmunol.178.10.6624 17475894

[29] Colonna-Romano G Bulati M Aquino A Pellicano M Vitello S Lio D. A Double-Negative (IgD-CD27-) B Cell Population Is Increased in the Peripheral Blood of Elderly People. Mech Ageing Dev (2009) 130(10):681–90. doi: 10.1016/j.mad.2009.08.003 19698733

[30] Pulendran B SA P O’Hagan DT. Emerging Concepts in the Science of Vaccine Adjuvants. Nat Rev Drug Discov (2021) 20(6):454–75. doi: 10.1038/s41573-021-00163-y PMC8023785 33824489

