# Supplementary figures and images for: Innate Immune Responses of Vaccinees Determine Early Neutralizing Antibody Production After ChAdOx1nCoV-19 Vaccination
Source: Front Immunol. 2022 Jan 25;13:807454. doi: 10.3389/fimmu.2022.807454 (PMC8822242; doi:10.3389/fimmu.2022.807454)

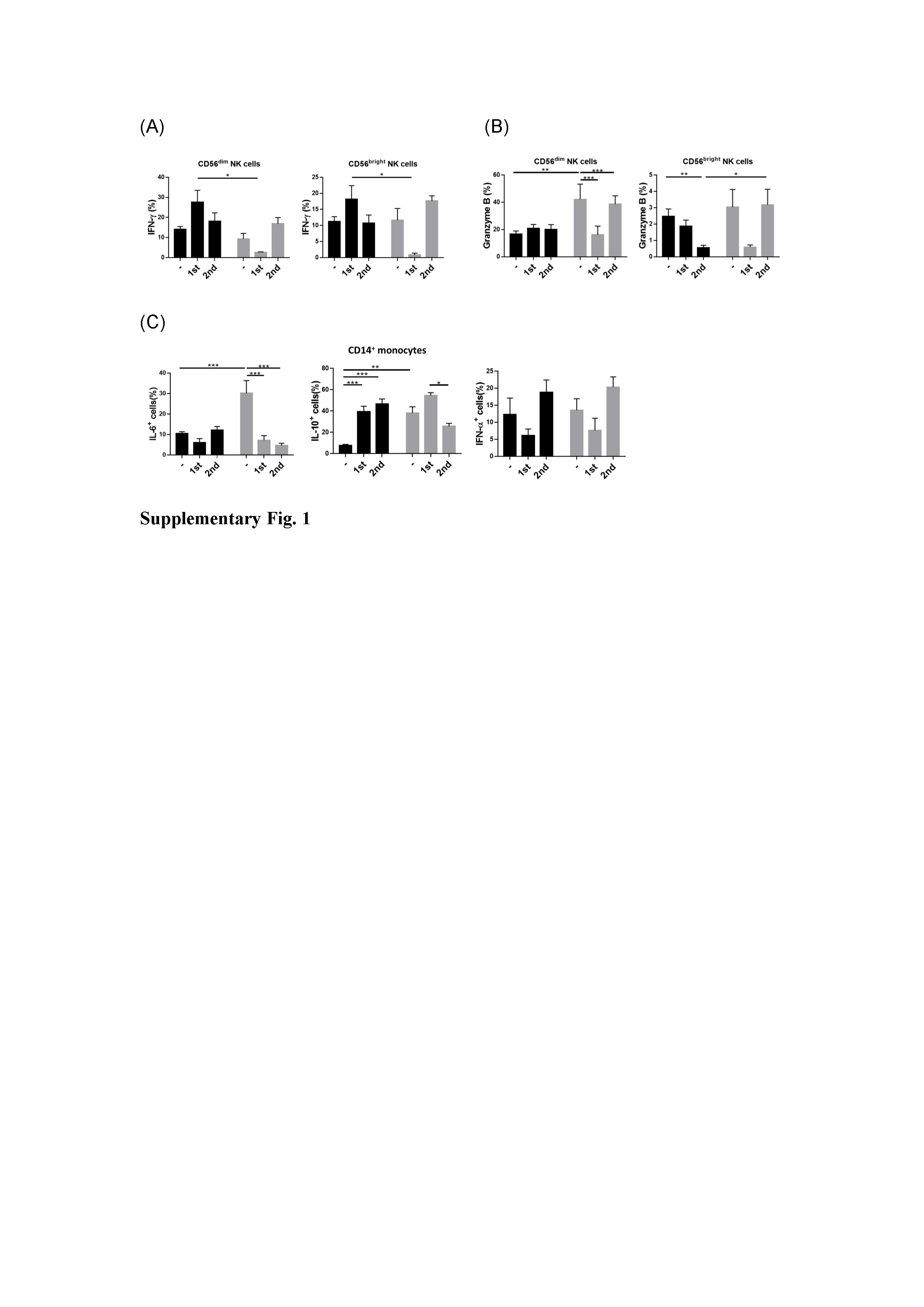

Supplement: Supplementary Figure 1 — Cytokine-producing NK and monocyte 3 days after ChAdOx1 nCoV-19 vaccination. PBMCs were isolated from young (black bars) and older adult vaccinees (gray bars) 3 days after the first and second vaccinations. The percentage of IFN-γ- (A) and granzyme B- (B) expressing cells in CD3-CD56dim or CD3-CD56brightNK cells; IL-6-, IL-10-, and IFN-α-expressing monocytes (C) of the young and older adult vaccinees were analyzed by flow cytometry. Statistical significance was determined using ANOVA with Tukey’s multiple-comparisons testing between all groups. (Black: young vaccinees; Grey: older adult vaccinees). (*p value < 0.05; **p value < 0.01; ***p value < 0.001). [file Image_1.jpeg]
