# Supplementary material for: Innate Immune Responses of Vaccinees Determine Early Neutralizing Antibody Production After ChAdOx1nCoV-19 Vaccination
Source: Front Immunol. 2022 Jan 25;13:807454. doi: 10.3389/fimmu.2022.807454 (PMC8822242; doi:10.3389/fimmu.2022.807454)
Supplement: Supplementary file 2 [file Table_1.docx]

**Supplementary Table 1**. Staining antibody and experiment product information

| **Product** | **Supplier** | **Catalogue Number** |
| --- | --- | --- |
| Poly (I:C) | InvivoGen | tlrl-pic |
| LPS-EB | InvivoGen | tlrl-eplps |
| Imiquimod-R837 | InvivoGen | Tlrl-imq |
| CpG | InvivoGen | tlrl-2006-1 |
| APC Mouse Anti-Human CD3 | BD Biosciences | 555342 |
| Alexa Fluor® 488 Mouse Anti-Human CD56 | BD Biosciences | 557699 |
| APC-Cy™7 Mouse Anti-Human CD16 | BD Biosciences | 557758 |
| APC-Cy™7 Mouse Anti-Human CD14 | BD Biosciences | 557831 |
| PE-Cy™7 Mouse Anti-Human CD68 | BD Biosciences | 565595 |
| BV421 Mouse Anti-Human MSR1 (CD204) | BD Biosciences | 742438 |
| BB700 Mouse Anti-Human CD86 | BD Biosciences | 566473 |
| FITC Mouse Anti-Human CD19 | BD Biosciences | 555412 |
| APC-H7 Mouse Anti-Human CD27 | BD Biosciences | 560222 |
| PE-Cy™7 Mouse Anti-Human IgD | BD Biosciences | 561314 |
| PE-Cy™7 Mouse Anti-Human IFN-γ | BD Biosciences | 560924 |
| Alexa Fluor® 700 Mouse Anti-Human Granzyme B | BD Biosciences | 561016 |
| FITC Rat Anti-Human IL-6 | BD Biosciences | 554544 |
| APC Rat Anti-Human IL-10 | BD Biosciences | 554707 |
| PE Mouse anti-Human IFN-α[2b] | BD Biosciences | 560097 |
| Protein Transport Inhibitor (Containing Brefeldin A) | BD Biosciences | 555029 |
| Perm/Wash Buffer | BD Biosciences | 554723 |
